# Intergovernmental Relations in the UK: Time for a Radical Overhaul?

**DOI:** 10.1111/1467-923X.12862

**Published:** 2020-06-14

**Authors:** Nicola McEwen, Michael Kenny, Jack Sheldon, Coree Brown Swan

**Keywords:** British politics, Brexit, devolution, territorial politics, intergovernmental relations

## Abstract

Brexit and the coronavirus pandemic have put relationships between the UK government and its devolved counterparts under growing strain. Tensions generated by both of these developments have exposed the inadequacies of the existing, under‐developed system for bringing governments together in the UK. The limitations of the current system include the *ad hoc* nature of intergovernmental meetings, and their consultative rather than decision‐making character. Drawing upon an analysis of how intergovernmental relationships are structured in five other countries, the authors offer a number of suggestions for the reconfiguration of the UK model. They explore different ways of enabling joint decision making by its governments, and argue against the assumption that England can be represented adequately by the UK administration. Without a serious attempt to address this dysfunctional part of the UK’s territorial constitution, there is every prospect that relations between these different governments will continue to deteriorate.

brexit has placed a huge strain upon different parts of the UK’s uncodified constitution. One of the most important areas where existing conventions and practices have been found wanting concerns relationships between the UK government and its devolved counterparts. Once an issue that mainly animated specialists, the absence of an adequate machinery of intergovernmental relations (IGR) within the UK has now become a question of great political sensitivity. At a time when the Scottish government has proposed a second independence referendum, and calls have been renewed for a border poll in Northern Ireland, the inadequacies of IGR could have a significant bearing on the future of the UK.

The Brexit process has exposed the absence of any agreed rules or procedures for enabling the UK’s four administrations to make decisions together. Reporting on the impact of Brexit on devolution, the House of Commons Public Administration and Constitutional Affairs Committee noted the ‘growing consensus that the current UK inter‐governmental relations mechanisms are not fit for purpose’.^1^ The committee highlighted the ineffectiveness of the Joint Ministerial Committee—the key forum for bringing together ministers from across the administrations—and an overreliance on informal, *ad hoc*, and often bilateral relationships. Calls for reform of IGR have also been made in recent years by the National Assembly for Wales Constitutional and Legislative Affairs Committee, the Scottish Parliament Finance and Constitution Committee, and the House of Lords Constitution Committee, among others.^2^

Leaving the EU has posed acute questions about whether European regulations should be replaced by UK‐wide regulatory frameworks, and how this is to be achieved in policy areas such as agriculture, the environment and fisheries, which fall within devolved competence. The EU’s legal and regulatory framework limited, though did not eliminate entirely, policy divergence between the constituent territories of the UK. But, without common frameworks, significant and disruptive forms of divergence across the UK’s own internal geography might emerge as each government makes policy in response to its own priorities, interests, and needs. Although divergence need not be problematic, some level of cooperation between the four administrations may be conducive to avoiding new barriers to trade and mobility across the UK. There are also issues of constitutional principle at stake. Devolved governments have stressed their constitutional right to preserve their own autonomy in policy areas considered to be within the legislative competence of the devolved institutions. This issue has already sparked sharp disagreements between the devolved governments on the one hand, and the UK government on the other, during the process of legislating for the UK’s EU exit.

Intergovernmental relations have also been under the spotlight in relation to the international trade deals that the UK government is keen to achieve, now that it has left the EU. Although trade is a reserved matter across all three devolution settlements, it is expected that the negotiating mandates and resulting deals will have an impact on devolved matters. In addition, the devolved governments are responsible for implementing any agreements which affect policy spheres within their jurisdiction. Each also feels that they have important sectoral interests to protect in future negotiations—for instance, the Scotch whisky industry or Welsh farming—and share the view that there needs to be a mechanism through which they can influence future negotiations. All have a stake in the negotiations over a future trade deal with the EU. This issue is especially acute for the Northern Ireland Executive, given the extent of cross‐border trade and mobility across the Irish border, now also the only land border between the UK and the EU.

The means by which the different governments of the UK relate to each other, and whether they can work together effectively as negotiations with the EU and third countries unfold, are now acutely important, and potentially incendiary, questions. They are made all the more difficult by the very different stances on Brexit that have been adopted by the UK and devolved governments. Whereas the May and Johnson governments were determined to deliver on the referendum mandate, the Scottish government took from the referendum a national mandate to oppose Brexit, or at least to seek the softest Brexit possible. And, although it had opposed Brexit in the referendum campaign, the Welsh government accepted the majority Leave vote in Wales, but similarly argued for a soft Brexit, wanting the UK to be closely aligned with the EU internal market. The collapse of the Northern Ireland Executive during the first phase of Brexit negotiations meant that it had neither a coherent position nor an institutional voice in them. The special arrangements set out in the Ireland/Northern Ireland protocol of the Withdrawal Agreement suggest that Northern Ireland will uniquely still be subject to EU single market regulations for goods. This poses further questions about whether, and to what extent, the restored executive could be a party to common UK regulatory frameworks, were they to be agreed. Moreover, the Scottish government’s ambition to seek a further referendum on independence, and the Johnson government’s firm rejection of such a demand, makes this an especially taxing political context, with deep mistrust between these governments.

There is an abiding need to recognise some of the main weaknesses in the existing machinery of IGR, and to try to reach consensus on what a more expansive and functional system of intergovernmental cooperation might involve. Some of the flaws in the current model were recognised by the governments themselves, when they instructed the secretariat of the Joint Ministerial Committee to undertake a joint review of IGR. This work was initiated in March 2018, but by the time the UK left the EU almost two years later, it had yet to produce any concrete proposals.

Drawing upon our analysis of how intergovernmental relations works in a number of other democratic states—specifically Australia, Belgium, Canada, Italy and Spain—we point below to a number of ideas that might offer help and inspiration for those tasked with reforming IGR in the UK context.^3^ The list of countries from which we draw insights includes formal federations and quasi‐federal systems, and incorporates a variety of models and practices. In Belgium, Italy and Spain, the machinery of IGR is heavily institutionalised, while in Australia and Canada it is more informal in character. In each of these cases, intergovernmental arrangements reflect the political and constitutional environment within which they have been established, but they nevertheless offer some useful insights for the UK. In the final section of the article, we argue that it is in the interests of all governments to improve intergovernmental relations within the UK, irrespective of the outcome of Brexit or the UK’s broader constitutional future.

## Flaws in the UK’s system of IGR

The idea of developing a coherent *system* of IGR has been, at best, an afterthought in the UK. The asymmetrical character of devolution in Scotland, Wales and Northern Ireland, and the absence of devolved government for England, militated against the task of devising structures of cooperative government. So too did the abiding assumption that devolution involved only the peripheries, not the central state itself. The Blair government’s devolution plans were highly centrifugal in character, neglecting almost entirely the questions of how policy interdependency would be managed, or power shared across administrations.

From the outset, ministers recognised the need for ‘good communication systems’ and a ‘non‐statutory machinery of cooperation’, but the emphasis was on informal, day‐to‐day interactions.^4^ The Joint Ministerial Committee (JMC) was established through the intergovernmental *Memorandum of Understanding* first published in 1999. However, it did not meet regularly for most of the first decade of devolution—except in its European format, where relevant ministers from each government met to coordinate ahead of European Council meetings. At a time when Labour was in office in the UK, day‐to‐day intergovernmental relationships in Scotland and Wales were mostly managed through informal political channels and existing relationships between leading figures in each government. As early as 2002, the House of Lords Constitution Committee expressed concern at ‘the sheer extent of the reliance on goodwill as the basis for intergovernmental relations within the United Kingdom’. It called for greater use of formal mechanisms, including the JMC, to help ensure that more robust processes would be put in place to cope with more challenging future circumstances, such as when governments in different parts of the UK would be led by different political parties.^5^

The need for an improved system for IGR became pressing only when this situation arose, following the formation of the first SNP Scottish government in 2007 and then the Conservative–Liberal Democrat coalition UK government in 2010. Some cosmetic changes to existing arrangements were introduced, but these made little difference to the overall effectiveness of the system. Within a few years, the new JMC sub‐forum to discuss ‘domestic’ issues, established in 2009, fell into disuse. Moreover, the trust required for any system of IGR to work properly evaporated in the run‐up to the 2014 Scottish independence referendum. The revisions to the Scottish devolution settlement in the wake of the referendum, which included new powers in otherwise reserved areas of tax and social security, brought new complications in their wake. Consequently, the cross‐party Smith Commission that had led the review of devolution called for the machinery of IGR to be reformed ‘as a matter of urgency and scaled up significantly’.^6^

In the wake of the Brexit referendum of 2016, there has been a considerable expansion in the use of IGR meetings in the UK. New forums emerged to discuss the implications of Brexit. Between its establishment in October 2016 and the UK’s formal exit from the EU in January 2020, a new sub‐committee of the JMC, the JMC (EU Negotiations), had met twenty times. Yet, the more frequent recourse to formalised structures of intergovernmental interaction has not necessarily made conversations between the different governments more productive or meaningful. Notwithstanding some notable examples of close cooperation between officials, ministers from the devolved governments have regularly complained that intergovernmental engagement, especially in ministerial forums, offers few opportunities for meaningful discussion or the constructive airing of differences. Privately, some ministers from the UK government also acknowledge the ineffectiveness of these meetings.^7^

While these weaknesses have been recognised by all governments, reaching agreement on a way forward has proved difficult so far. Only the Welsh government has thus far presented clear proposals for a reformed structure of IGR. This entailed a fairly radical vision of an enhanced ‘UK Council of Ministers’, modelled loosely on the EU Council of Ministers, where decisions would be made by consensus or, where that was not possible, by qualified majority voting.^8^ The UK government remains wary of a model that might constrain its decision‐making authority or place it on a par with other devolved administrations. For its part, the Scottish government is reluctant to be drawn into a model of intergovernmental cooperation that might constrain its autonomy and detract from the case for independence. For Northern Ireland, UK IGR is only one strand of the delicate balance of east‐west and north‐south institutional relationships secured by the Belfast/Good Friday Agreement. Although unionists may welcome a stronger system of cooperative governance in the UK, this might well generate unease among nationalists, especially if it was seen to undermine the importance of the north‐south dimension.

If reforms are to achieve a more functional and transparent system of IGR, they would need to address some of the gaps and flaws in the current model. We make the case specifically for: (i) a clearer articulation of the principles which underpin IGR; (ii) some concrete changes to enhance the effectiveness of the JMC; and (iii) consideration of options for how England might be brought into the intergovernmental fold. In making these arguments we draw upon comparative research into intergovernmental relations within other countries. While there is no single template to recommend in this area, there are lessons to be learned, and insights gained, from those states that have developed more institutionalised modes of intergovernmental working.

## Principles

In multilevel political systems, it is common for IGR arrangements to be underpinned by a set of core principles to which all the relevant governments broadly assent. These are sometimes articulated explicitly in the constitution, in statute or elsewhere (as in Australia, Belgium, Italy and Spain), and sometimes they are left implicit (as in Canada). Principles for intergovernmental working can be quite practically focussed, or more broad‐brush and aspirational. In the UK, the most recent version of the *Memorandum of Understanding* agreed by the UK and devolved governments makes reference to certain basic, shared principles, including good communication and information exchange, the supremacy of the UK Parliament, and the importance of the Sewel convention (under which the UK Parliament ‘would not normally legislate with regard to devolved matters expect with the agreement of the devolved legislature’).^9^

These are very broad principles and tend to be interpreted very differently by the various parties involved. For example, what amounts to ‘good’ communication and what might be ‘practicable’ in terms of information exchange are matters of (often diverging) judgement. The ambiguity of the Sewel convention has been thrown into relief by the recent experience of Brexit legislation passed in the UK Parliament without devolved consent, despite its significance for devolved competences. The European Union (Withdrawal) Act 2018 was passed without the consent of the Scottish Parliament, and the European Union (Withdrawal Agreement) Act 2020 passed despite all three devolved legislatures withholding their consent.

The *Memorandum of Understanding* was last updated in 2013 in a very different political context, prior to the Scottish independence and Brexit referendums. There have since been numerous calls to revise and reinforce the principles that undergird IGR, and various failed attempts to secure the agreement of all governments. The starting point for any revised set of principles might be for each government to reaffirm its commitment to respecting the constitutional status and democratic mandate of the other levels of government. Other fundamental principles may include an explicit recognition that the reality of modern government in a multilevel political system requires some degree of intergovernmental cooperation. Areas where there is clear interdependence between reserved and devolved powers might be identified as suitable subjects for cooperation. This may include many of the powers repatriated from the EU and hitherto subject to EU regulation that fall within devolved competence, such as in relation to agriculture and environmental standards. But, from the perspective of the devolved governments, it may also include areas of reserved competence, including trade and competition policy, that have an impact on devolved matters. A principle that recognises policy interdependency might be accompanied by a further, related, principle of proportionality—that common intergovernmental approaches, mechanisms and forums be established only when necessary to achieve a mutually agreed purpose. This should ensure that IGR does not place an excessive burden on the time and resources of governments, and that it does not have a debilitating effect on the authority of participating administrations.

Any enhanced system of intergovernmental coordination or codecision raises important issues about accountability. IGR processes are inherently executive‐dominated and often conducted behind closed doors, but the lack of transparency of the UK’s model has been especially notable. Minutes, where they are taken, are generally not published. The JMC ‘annual report’ is not always published annually, and the level of detail it provides is typically limited to dates of meetings, agenda items, and notifications of disputes invoked under the dispute protocol. Neither the post‐meeting communiqués nor the annual report offer much insight into the substance of discussions or disputes. The lack of transparency in the system has been one of the most frequently expressed concerns of parliamentary committees. It constrains the ability of parliaments and the electorate to hold governments to account, and to judge between competing accounts and interpretations of the conduct of intergovernmental meetings. The implications for democratic accountability may become more pronounced if the use and functions of intergovernmental forums increase. There is, of course, a need for ministers and officials to have a safe private space to share information and policy discussions, but a principled commitment to greater transparency might counterbalance the risks that any system of enhanced cooperative governance could weaken the democratic accountability of each administration.

Debating matters of principle might well feel irrelevant or unrealistic, given the chasm that divides the Scottish and UK governments in particular on many questions, but some agreement on shared principles could generate greater clarity about the purposes and operation of IGR. Where agreements on principle are difficult to reach, steps can still be taken to develop forums and practices that could support more effective cooperation and deliberation between governments.

## Strengthening the JMC

When the UK’s system of IGR has previously been evaluated, emphasis has usually been placed on the weaknesses of the JMC structure, where most of the formal aspects of intergovernmental working happen. There have been criticisms of the *ad hoc* nature of these meetings, the dominant role taken by the UK government in organising and chairing them, and the tendency for them to descend into the airing of grievances and accusations rather than promoting productive policy discussions. The limited nature of the JMC’s role, focussing on consultation rather than decision making, and the inadequacy of existing dispute resolution arrangements, have also been widely criticised.

JMC meetings are currently arranged on an *ad hoc* basis. They are often called at short notice and there have been long periods of unexplained inactivity. This could be easily avoided by agreeing a schedule of dates for future meetings in advance, as happens in Australia, Belgium and Italy. Agreeing a schedule would help to establish these meetings as a regular and important feature of the political calendar. Equally, routinely rotating chairs and meeting venues between the governments, something which has recently started to happen in Brexit‐related forums, could be an important symbolic change.

The UK’s JMC structure has included few forums that provide for regular meetings of sectoral ministers. By contrast, Belgium, Canada and Spain each have more than thirty intergovernmental forums and Australia has eight sectoral ‘COAG Councils’, plus other inter‐ministerial bodies. Such a proliferation of forums might not itself be desirable but the emergence over recent years of more policy areas where competences span the reserved and devolved spheres suggests that more sectoral forums could be useful in the UK context. This is already happening in some areas, but current practice is *ad hoc* and patchy. For example, a Ministerial Forum on Trade first met in January 2020 to provide for devolved input into the process of negotiating post‐Brexit trade deals.

The Brexit process and the challenges to UK governance that it has generated lend support to the idea that it may be time to shift the remit of the JMC beyond consultation to codecision. The current *Memorandum of Understanding* explicitly states that the JMC is ‘not a decision‐making body’, but merely a consultative one. This is linked to the devolution statutes, each of which has sought to establish a clear distinction between matters that are reserved exclusively to the UK Parliament and matters that are principally for the devolved institutions to determine, Westminster parliamentary sovereignty notwithstanding. However, these distinctions have become less clear, as new powers in areas such as tax and welfare have been partially devolved, and as previously EU policy competences have been repatriated.

In most other federal and quasi‐federal systems, IGR forums play a key role in concluding intergovernmental agreements in areas where central and sub‐state competences intersect. Most of these systems ensure cross‐governmental working in the policy process from an early stage. In Belgium, where entrenched tensions between linguistic groups make intergovernmental politics particularly sensitive, consultation takes place from the early stages of policy development at official level, and also between ministers and, if necessary, heads of government. This has ensured that, even during periods of general political instability, agreements have been reached on a regular basis. This kind of bottom‐up approach to IGR has been used with some success in the UK during the process of developing post‐Brexit common frameworks, mainly in technical areas. But it may be even more expedient in politically salient areas where proposed policies cut across or affect the competences of different administrations, as would be the case, for example, with fisheries management or state aid.

If joint decision making through the JMC or a similar forum were to be attempted, it would be necessary to establish some ground rules, and this is not a straightforward task. The Welsh government report suggested a form of qualified majority voting where consensus could not be reached, whereby the consent of the UK government and at least one devolved government would be sufficient for a decision to be made. None of the other administrations have embraced this proposal and it would likely be met with considerable resistance.

In most of the systems we examined, the expectation is that consensus will ultimately emerge following negotiations. This means that decisions made through IGR processes do not result in policies or regulatory requirements being imposed on one or more government without their consent, in areas that fall within their competence. Where it is impossible to reach consensus on a proposed agreement, those governments that wish to proceed can often do so, with others opting out. An ‘opt‐out’ system operates among Canadian governments and there is also experience of this in Australia, for example, when Western Australia opted out of a controversial healthcare agreement in 2010. Alternative procedures that risk imposing outcomes in the face of opposition are likely to generate conflict and new grievances, and lead to a further erosion of trust.

A glaring gap in the UK’s current system is the lack of a satisfactory procedure for resolving conflict. A *Protocol for Avoidance and Resolution of Disputes* was agreed by the UK and devolved governments in 2010.^10^ Five official disputes have been raised under the protocol, although only one—whether spending on the 2012 Olympic Games in London should be treated as ‘English’ spending for the purposes of the Barnett formula—was escalated to a formal JMC disputes panel under the protocol. Since then, the dispute resolution protocol has come in for increasing criticism from the devolved governments and parliamentary committees. The criticisms levelled at it have focussed particularly on the provision that disputes panels are chaired by a UK government minister, making it difficult for the devolved governments to have confidence that their cases would be considered fairly. Former Welsh First Minister, Carwyn Jones, asked how the Welsh government could ever trust a procedure that ‘leaves the final decision‐making responsibilities with the UK government, empowered in what we might call its “federal” capacity, to mark its own homework’.^11^

A dispute resolution process chaired by one of the parties to the dispute is out of step with the practices of other countries with multilevel systems of government. Some of these make provisions for an independent panel to become involved in certain categories of dispute. For example, the recent Canadian federal–provincial agreement on internal trade includes provisions for an independent arbitration panel. In Belgium there is a standing dispute resolution procedure involving an *ad hoc* tribunal presided over by a magistrate. These are regarded as a last resort and are not often used, but they provide the governments with some reassurance that they have recourse to a fair process in the event that negotiation fails. A revised dispute resolution protocol for the UK could provide for independent intervention in a dispute, where requested by the disputing parties. Rather than an arbitrator making a legal ruling that bound all of the relevant parties, a suitably qualified mediator would be more in keeping with the relatively informal nature of UK governance.

## Representing England

A more effective system of intergovernmental relations that can command confidence from all parts of the UK may require consideration of how England’s interests can be more explicitly represented. In the UK’s highly asymmetrical devolution arrangements, the UK government claims to speak for the entirety of the UK while also, simultaneously, being required to speak for England as a territorial entity. This may be increasingly untenable. From the perspective of the devolved governments, the ‘dual‐hatted’ role performed by the UK government is problematic because of the worry that England’s interests may be the primary concern of the largest government of all. Conversely, various commentators and campaigners have suggested that the absence of separate English representation denies England the sort of national voice on important policy issues afforded to the devolved areas. Meanwhile, the leaders of some of England’s largest combined authorities, and the Mayor of London, Sadiq Khan, have argued that the current system of IGR cuts them out from the kind of top‐table influence they deserve.^12^ These are difficult issues to resolve in the absence of wider constitutional reforms that aim to achieve more symmetrical constitutional arrangements. Nevertheless, an increase in the scope and significance of IGR resulting from Brexit would generate a strong case for considering more deeply how England might be represented appropriately within these processes.

One potential model would involve reforming how the UK government participates in them, so that the distinction between its UK and English roles became clearer. This might be achieved by the appointment of a Minister for England, with responsibilities including representing English interests in IGR. A Minister for England appeared in Labour’s general election manifesto in 2017 (although this proposal was dropped in 2019). Alternatively, a relevant sectoral minister could be tasked with representing English interests when appropriate, while another UK government minister would also attend with a focus on seeking the best outcome for the UK as a whole. This sort of approach could be particularly well suited to forums dealing with issue areas like agriculture and fisheries, where the material interests of the English sectors are distinct from those in the devolved territories.

An alternative, or complementary model would be to provide an institutionalised opportunity for regional leaders in England to feed into IGR discussions. Combined authorities with directly‐elected mayors have now been established in eight, mostly urban, areas in England, with the potential for more to emerge over the next few years. Combined authority mayors, together with some local government representatives, could participate in an English Leaders’ Forum convened by the UK government to focus on matters relevant to English sub‐national government, such as regional funding, further devolution of powers, and the regulation of the internal market in areas where devolution has taken place. Were such meetings scheduled to align with a more routinised schedule of JMCs, it could provide an opportunity for regional leaders to have an input into the UK government’s representation of English interests within the JMC. The Conference of State, Cities and Localities organised along these lines in Italy could provide an interesting point of reference for such a reform.

## IGR and the future of the UK

As debates intensify over the constitutional futures of Scotland and Northern Ireland, it may seem a little quixotic to stress—as we do—the importance of a little known, barely visible system of meetings which bring together the different administrations that together govern the UK. However, improving relationships between the UK's network of governments could do much to mitigate territorial tensions, as well as making an important contribution to the legitimacy and functionality of governance across the UK. In the short to medium term, a system of joint decision making may support the UK’s prospective departure from the EU’s regulatory regimes. As powers come back from Brussels, there is greater risk of significant internal disruption to the workings of the UK’s economy without cooperation and at least some coordination in areas such as animal health, food labelling and trade.

The need for more cooperative and productive relationships between different tiers of government has been further highlighted by the ongoing COVID‐19 pandemic which has brought the UK, and the world, to a near standstill in the early months of 2020. In this emergency situation, intergovernmental working and coordination have been taking place within the context of the Civil Contingencies (COBR) and associated Cabinet Sub‐Committees, and not within the JMC structure where they normally happen. This major crisis has thrown into sharp relief the importance of productive working relationships between all levels of government, and has also reminded politicians and publics of some of the profound, shared interests that continue to exist within the UK—and would do so whatever the constitutional arrangements between these territories. It is in the interests of all governments in the UK—despite their considerable political differences—to work together to devise a set of procedures that would help them make better decisions in concert as and when necessary.

A revised and enhanced model of IGR could contribute to the development of a more satisfactory and legitimate system of cooperative decision making across governments. In pretty much every other Western democracy that includes significant powers for smaller geographical units within its borders, such a system exists. This does not prevent territorial disputes from arising, but clearer principles, procedures and practices can help to mediate them when they do emerge. Brexit poses significant challenges to the UK’s territorial governance. Without addressing the widely acknowledged flaws in the UK’s system of intergovernmental relations, there is very little to stop disagreements between the constituent governments of the UK escalating into the kinds of open and deep conflicts that may well push it nearer to the point of breakup. Finding ways to work together more effectively is not just desirable within the current constitutional framework. Even in the event of Scottish independence, or Irish reunification, various kinds of cooperation will still be necessary, by virtue of geography and the continued interdependence of the economies and societies that coexist on these islands. Functioning mechanisms of IGR within the union could also provide a model for relations outside it.

## Notes

^1^
House of Commons Public Administration and Constitutional Affairs Committee, *Devolution and Exiting the EU: Reconciling Differences and Building Strong Relationships?*, HC 1485, 2018, p. 38.

^2^
House of Lords Constitution Committee, *Inter‐Governmental Relations in the United Kingdom*, HL Paper 146, March 2015; Scottish Parliament Finance and Constitution Committee, *European Union (Withdrawal) Bill LCM—Interim Report*, SP Paper 255, January 2018; Senedd Constitutional and Legislative Affairs Committee, *UK Governance Post‐Brexit*, February 2018.

^3^
This section of the article draws upon the report prepared by the authors for the Joint Review initiated by the JMC; see N. McEwen, M. Kenny, J. Sheldon and C. Brown Swan, *Reforming Intergovernmental Relations in the United Kingdom*, 2018; https://www.centreonconstitutionalchange.ac.uk/publications/reforming‐intergovernmental‐relations‐united‐kingdom (accessed 19 May 2020).

^4^
Lord Sewel, Parliamentary Under‐Secretary of State, Scottish Office, *House of Lords Debates*, vol. 590, 17 June 1998, col.1567–1573.

^5^
House of Lords, Select Committee on the Constitution, *Devolution: Inter‐Institutional Relations in the United Kingdom*, 2002; https://publications.parliament.uk/pa/ld200203/ldselect/ldconst/28/28.pdf (accessed 19 May 2020).

^6^
The Smith Commission, *Report of the Smith Commission for Further Devolution of Powers to the Scottish Parliament*, 27 November 2014; https://webarchive.nationalarchives.gov.uk/20151202171029/http://www.smith‐commission.scot/wp‐content/uploads/2014/11/The_Smith_Commission_Report‐1.pdf (accessed 19 May 2020).

^7^
Anonymous interviews with former UK government ministers conducted by the authors.

^8^
Welsh Government, *Brexit and Devolution: Securing Wales’ Future*, 2017; https://gov.wales/sites/default/files/2017‐06/170615‐brexit%20and%20devolution%20(en).pdf

^9^
UK Government, Scottish Government, Welsh Government and Northern Ireland Executive, 2013, *Devolution: Memorandum of Understanding and Supplementary Agreements*. https://assets.publishing.service.gov.uk/government/uploads/system/uploads/attachment_data/file/316157/MoU_between_the_UK_and_the_Devolved_Administrations.pdf (accessed 19 May 2020).

^10^
UK Government, Scottish Government, Welsh Government and Northern Ireland Executive, 2010, *Protocol for Avoidance and Resolution of Disputes,* 2010; https://assets.publishing.service.gov.uk/government/uploads/system/uploads/attachment_data/file/62121/avoidance‐resolution.pdf (accessed 19 May 2020).

^11^
C. Jones, ‘Brexit and devolution: stresses, strains and solutions’, speech at Institute for Government, 10 September 2018.

^12^
Public Administration and Constitutional Affairs Committee, Oral evidence: Devolution and Exiting the EU, HC 484 2017–19, 5 February 2018, Q202; 26 June 2018, Q889–891.

